# Computational tools for clinical support: a multi-scale compliant model for haemodynamic simulations in an aortic dissection based on multi-modal imaging data

**DOI:** 10.1098/rsif.2017.0632

**Published:** 2017-11-08

**Authors:** Mirko Bonfanti, Stavroula Balabani, John P. Greenwood, Sapna Puppala, Shervanthi Homer-Vanniasinkam, Vanessa Díaz-Zuccarini

**Affiliations:** 1 Department of Mechanical Engineering, University College London, Torrington Place, London WC1E 7JE, UK; 2 Leeds Institute of Cardiovascular and Metabolic Medicine, University of Leeds, Leeds LS2 9JT, UK; 3 Leeds Teaching Hospitals NHS Trust, Leeds LS1 3EX, UK; 4 University of Warwick Medical School & University Hospitals Coventry and Warwickshire NHS Trust, Coventry CV2 2DX, UK

**Keywords:** patient-specific simulation, aortic dissection, computational fluid dynamics, fluid–structure interaction, Windkessel model, moving boundary

## Abstract

Aortic dissection (AD) is a vascular condition with high morbidity and mortality rates. Computational fluid dynamics (CFD) can provide insight into the progression of AD and aid clinical decisions; however, oversimplified modelling assumptions and high computational cost compromise the accuracy of the information and impede clinical translation. To overcome these limitations, a patient-specific CFD multi-scale approach coupled to Windkessel boundary conditions and accounting for wall compliance was developed and used to study a patient with AD. A new moving boundary algorithm was implemented to capture wall displacement and a rich *in vivo* clinical dataset was used to tune model parameters and for validation. Comparisons between *in silico* and *in vivo* data showed that this approach successfully captures flow and pressure waves for the patient-specific AD and is able to predict the pressure in the false lumen (FL), a critical variable for the clinical management of the condition. Results showed regions of low and oscillatory wall shear stress which, together with higher diastolic pressures predicted in the FL, may indicate risk of expansion. This study, at the interface of engineering and medicine, demonstrates a relatively simple and computationally efficient approach to account for arterial deformation and wave propagation phenomena in a three-dimensional model of AD, representing a step forward in the use of CFD as a potential tool for AD management and clinical support.

## 1. Introduction

Computational fluid dynamics (CFD) has provided significant insight into the haemodynamics of many cardiovascular pathologies, being particularly amenable to study aortic dissection (AD) due to its complexity and patient-specific nature [1]. AD is characterized by the separation of the layers of the aortic wall. A tear in the intima layer allows blood to flow within the aortic wall inducing the formation of two flow channels, the true (TL) and false lumen (FL), separated by an intimal flap (IF) [2]. Diagnosis, management and treatment of AD are patient specific and difficult; experts claim that ‘difficulty in diagnosis, delayed diagnosis or failure to diagnose are so common as to approach the norm for this disease, even in the best hands…’ [3]. Initial management of acute AD focuses on pain control, heart rate and blood pressure management, followed by surgical intervention, typically involving stenting of the entry tear.

Although *type B dissections* (i.e. AD involving only the descending aorta) have lower initial mortality than *type A* (i.e. AD of the ascending aorta), they carry a poor long-term prognosis, with late-term complications reported in 20–50% of cases within 5 years [4].

Detailed characterization of complex intra-aortic haemodynamic parameters via CFD, which currently cannot be determined *in vivo*, has the potential to aid clinical decision-making around AD; for instance, by identifying those prone to adverse outcomes and supporting clinicians by simulating different interventional strategies [5–7].

A number of computational studies on AD have been published in the last decade [8]. CFD modelling approaches differ significantly across studies, especially regarding the boundary conditions (BCs) and the treatment of the wall. As the simulation of the whole vascular system is unpractical and patient specific, time-varying pressure and flow waveforms at all termination branches are usually not available, different strategies have been developed, e.g. via the coupling of zero-dimensional (0D) Windkessel models to the outlets of a three-dimensional (3D) domain, forming together a multi-scale model (referring to the combination of models of different dimensions) representing the vascular system [9].

However, this coupling is relatively challenging, needing to be handled appropriately and Windkessel parameters must be accurately tuned. With the exception of a few studies [2,5], most often simpler BCs are adopted in AD studies, such as flow-split [10], constant zero-pressure [11] or pressure waveforms taken from the literature [12].

High complexity and the computational demands of fluid–structure interaction (FSI) simulations means a rigid-wall approximation is widely used, neglecting the effects exerted on the fluid flow by the vessel wall motion and vice versa. In FSI, high uncertainties regarding the wall thickness and mechanical properties of the dissected vessel remain [13], complicating their application to AD studies. Nonetheless, recent FSI work by our group [14] showed that wall motion has an impact on clinically relevant haemodynamic parameters for AD, and thus should be accounted for. Although a reasonable match between computational and experimental data has been found in recent attempts to validate simulation results against *in vivo* phase-contrast magnetic resonance imaging (PC-MRI) [5,11], discrepancies attributed to a rigid-wall assumption remained. Hence, the need for reliable *compliant* models of AD is evident.

This study aims to tackle some of these challenges through the development of a novel framework for patient-specific simulations, combining multi-scale and compliant AD models as well as dynamic BCs. A flexible and robust method for tuning Windkessel parameters using non-invasive patient-specific clinical data is presented. Moreover, the rigid-wall assumption is addressed through the development of a novel and computationally efficient approach to account for the vessel wall motion in CFD simulations. Simulation results are validated against a rich and unique patient-specific clinical dataset from multiple imaging modalities such as two-dimensional (2D) PC-MRI, 2D cine-MRI and computed tomography (CT) scans.

## 2. Material and methods

### 2.1. Patient information

**Figure 1.**
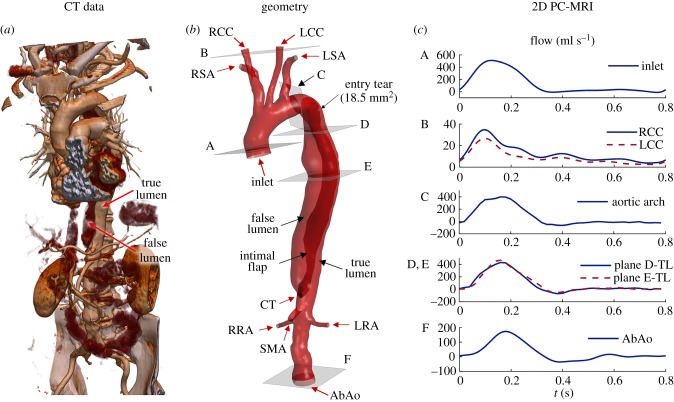
(*a*) Rendering of the CT data showing the dissected aorta. (*b*) Three-dimensional model extracted from CT data representing the dissected aorta from the ascending aorta (inlet) to the abdominal aorta (AbAo). The supra-aortic branches (left and right subclavian arteries (LSA, RSA), left and right common carotid arteries (LCC, RCC)) and the main visceral branches (coeliac trunk (CT), superior mesenteric artery (SMA), left and right renal arteries (LRA, RRA)) are included. (*c*) Flow-rate curves at different locations extracted from 2D PC-MRI data: (A) ascending aorta; (B) RCC and LCC; (C) aortic arch, distal to the LSA; (D,E) flow rate in the TL of the dissected aorta at two sections along the descending aorta, above and below the diaphragm, respectively; (F) AbAo, proximal to the iliac bifurcation.

Data from a 77-year-old man with a chronic Stanford type B AD were studied. The dissection originated approximately 40 mm distal to the left subclavian artery, extended throughout the length of the descending aorta and terminated about 10 mm distal to the coeliac trunk. From CT scans (figure 1*a*), one entry tear (area approx. 18.5 mm^2^) was located approximately 10 mm distal to the proximal end of the dissection; no other communication between the TL and FL was evident from CT data, confirmed by a reduced flow in the FL observed in PC-MRI data.

### 2.2. Clinical dataset

#### 2.2.1. CT scans

The entire aorta was imaged with a 16-slice CT scanner (Sensation 16; Siemens AG, Munich, Germany; 120 kV, 380 mA s, rotation time: 0.5 s, field of view (FOV): 284 mm, slice thickness: 1 mm, reconstruction kernel: B20F, contrast agent: Ultravist 300; Bayer AG, Leverkusen, Germany) obtaining 946 slices with in-plane resolution = 0.55 mm and inter-slice distance = 0.7 mm (figure 1*a*).

The geometry of the dissected aorta was extracted using semi-automated segmentation tools based on thresholding, implemented in ScanIP (Synopsys Inc., CA, USA). Smoothing operations were used on the resulting mask to reduce pixellation artefacts. The IF separating the FL from the TL was identified based on greyscale-value differences in the two lumina; however, because it was difficult to clearly resolve its thickness from CT images, it was approximated as a zero-thickness membrane for modelling purposes. The aortic branches were cropped perpendicularly to the vessel's longitudinal axis to provide a flat surface at the boundaries. The resulting geometry is shown in figure 1*b*, representing the dissected aorta: from the ascending aorta, just distal to the sinotubular junction of the aortic root, to the abdominal aorta (AbAo) just proximal to the iliac bifurcation [15].

#### 2.2.2. MRI data

High spatial and temporal resolution electrocardiogram (ECG)-gated cine-imaging sequences (2D cine-MRI; slice thickness: 10 mm, repetition time: 3.1 ms, echo time: 1.5 ms, FOV: 375 mm, in-plane resolution: 1.1 mm, temporal resolution: 40 phases/cardiac cycle, steady-state free precession) were acquired with an Ingenia 1.5 T MRI scanner (Philips Healthcare, Amsterdam, The Netherlands) at several sections perpendicular to the aorta (indicated in figure 1*b*). Additional slices (coronal plane) were acquired to detect IF motion in the distal part of the dissection.

Additionally, through-plane phase-contrast velocity mapping images (2D PC-MRI) were acquired at the same locations for flow quantification (slice thickness: 6 mm, repetition time: 17 ms, echo time: 1.9 ms, FOV: 400 mm, in-plane resolution: 1.56 mm, temporal resolution: 40 phases/cardiac cycle, velocity encoding: 200 cm s^−1^).

At each anatomical location, a single slice was acquired, for each of the two pulse sequences. The total acquisition time for the whole MRI protocol was approximately 80 min.

The lumen cross-sectional area (*A*) was quantified from 2D cine-MRI sequences using thresholding algorithms available in ImageJ (NIH, MD, USA). The normalized cross-sectional area variation (ΔA*) was calculated as2.1
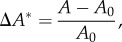
where *A*_0_ is the minimal cross-sectional area measured during a cardiac cycle.

Velocity information was extracted from the PC-MRI sequences using the GTFlow software (GyroTools LCC, Zürich, Switzerland); flow-rate curves measured at different sections are reported in figure 1*c*. Flow curves A and B are used as BCs, whereas C–F are used for validation. A heart rate of 75 bpm and a stroke volume of 107.6 ml were reported, corresponding to a cardiac output of 8.1 l min^−1^.

### 2.3. Computational simulation set-up

#### 2.3.1. Flow model and boundary conditions

The Navier–Stokes (NS) and continuity equations for 3D time-dependent flows were solved with finite-volume-based CFD solver ANSYS-CFX 17.0 (ANSYS Inc., PA, USA).

**Figure 2.**
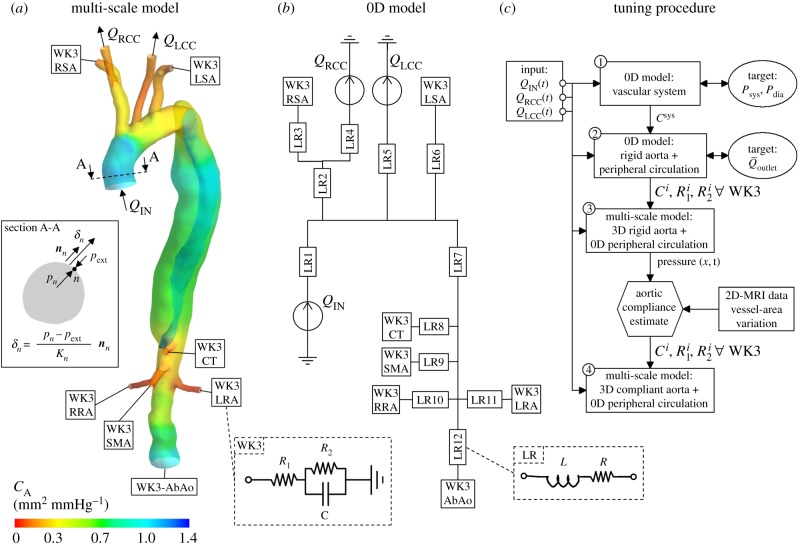
(*a*) Schematic of the multi-scale model domain and its BCs. WK3 indicates the Windkessel models coupled at the outlets (see inset). The colour map shows the area compliance of the aorta (*C*_A_ = Δ*A*/Δ*P*), estimated with patient-specific 2D cine-MRI data. The inset shows a cross-section of the ascending aorta and a schematic depicting how the displacement of a node *n* on the vessel wall is related to the pressure *p_n_* calculated in the fluid domain according to the MB method. (*b*) Schematic of the 0D model (lumped-parameters model) used during the tuning procedure. LR indicates the elementary building block, composed by an inertance (*L*) and a resistance (*R*) (see inset), representing a vessel segment: LR1: ascending aorta and arch, LR2: brachiocephalic trunk, LR3: right subclavian artery, LR4: right common carotid, LR5: left common carotid, LR6: left subclavian artery, LR7: descending aorta, LR8: coeliac trunk, LR9: superior mesenteric artery, LR10: right renal artery, LR11: left renal artery, LR12: abdominal aorta. (*c*) Flowchart of the procedure adopted to tune the parameters of the multi-scale model. Steps 1–4 are described in more detail in the text.

Blood was modelled as incompressible with density = 1056 kg m^−3^ and non-Newtonian viscosity described by the Carreau–Yasuda model with parameters taken from Gijsen *et al*. [16]. Blood flow was considered as laminar, a common assumption in large arteries [2,12]. BCs applied to the fluid boundaries are shown schematically in figure 2*a*. In particular, uniform velocity profiles were prescribed at the inlet and LCC and RCC boundaries for which patient-specific flow-rate waveforms were available from PC-MRI data (i.e. *Q*_IN_(*t*), *Q*_LCC_(*t*), *Q*_RCC_(*t*), figure 1*c*). The corresponding mean Reynolds and Womersley numbers, based on the inlet diameter of the reconstructed aorta, were equal to 1408 and 23, respectively. The peak Reynolds number was 5275, which is below the critical Reynolds number for transition to turbulence, based on a dynamic viscosity of 4 × 10^−3^ Pa s and following Peacock *et al*. [17].

The remaining outlets of the 3D model were coupled to three-element Windkessel models (WK3s), shown as electrical analogues in figure 2*a*, inset. The flow curve obtained from PC-MRI at the AbAo was not applied as the outflow BC to avoid constraining the wave propagation. The flow (*Q*) and the mean pressure (*P*) over these boundaries are related by2.2
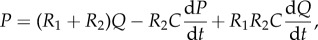
where *R*_1_ and *R*_2_ represent the proximal and distal resistances, and *C* is the compliance of the distal vasculature. *R*_1_ is used to absorb the incoming waves and reduce artificial wave reflections, as shown by Alastruey *et al*. [18]. WK3s seem to be the best compromise among other physiologically relevant 0D outflow models to simulate the peripheral vasculature [19], and should be used—instead of purely resistance models—when a significant compliance is located in the modelled distal vasculature [18]. WK3 parameters must be tuned to obtain physiological flow/pressure waveforms. The tuning procedure is described in §2.3.3.

Pressure at the model outlet is updated at every solver loop according to equation (2.2), using as input the current flow rate calculated at the outlet of the 3D domain. Derivative terms were discretized with a first-order backward Euler approach using flow and mean pressure values calculated over the boundary at the previous time step.

A no-slip condition was applied; the IF was assumed to be rigid. The external aortic wall was allowed to expand and contract with pressure fluctuations using a moving boundary (MB) technique accounting for aortic compliance, as described in §2.3.2.

The NS equations were spatially and temporally discretized with a high-resolution advection scheme [20] and a second-order implicit backward Euler scheme, respectively, using a uniform time step of 1 ms, good enough for time-step size-independent results.

#### 2.3.2. Moving boundary method

An MB algorithm was employed to account for the compliance of the aorta, capturing wave transmission effects. Details are presented in Bonfanti *et al*. [21] and summarized here. The motion of the ascending aorta due to the heart motion has been disregarded.

Assuming that the displacement of the aortic wall follows the local surface-normal direction and is linearly related to the fluid pressure (figure 2*a*, inset), the displacement *δ_n_* (m) of each mesh node *n* on the external vessel wall is calculated as2.3
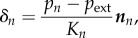
where *p_n_* (Pa) is the pressure at node *n*, *p*_ext_ (Pa) is the external pressure set as equal to the mean diastolic pressure over the aortic wall (in this study 76 mmHg), ***n****_n_* is the unit normal vector in the outward direction and *K_n_* (N m^−3^) is a measure of the wall stiffness at the location of node *n*. Under the hypothesis of a circular cross-section, *K_n_* can be related to the vessel area compliance *C*_A_ as follows:2.4
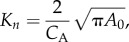
where *A*_0_ is the vessel cross-sectional area at the location of node *n*. *C*_A_ can be estimated using patient-specific 2D cine-MRI sequences, as described in §2.3.3.

Mesh displacement equations were solved so as to obtain an implicit two-way coupling between mesh motion and fluid dynamics.

#### 2.3.3. Model tuning based on patient-specific data

The parameters of the combined 3D aortic model and 0D WK3 models were specifically tuned for the simulated patient. *R*_1_, *R*_2_ and *C* were specified for each WK3 to characterize the peripheral circulation, while *C*_A_ needed to be estimated to mimic the compliance of the aortic model.

The aim of the tuning procedure was to obtain physiological values for the mean flow rate at the outlets 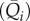, and target systolic (*P*_sys_) and diastolic (*P*_dia_) blood pressure at the inlet.

Patient-specific brachial *P*_sys_ and *P*_dia_ (150 and 80 mmHg, respectively) were used as pressure *target* values. *Target* values for 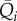 were set based on available PC-MRI and literature data, as summarized in table 1.

**Table 1.** Mean blood flow at the aortic branches used for tuning the Windkessel parameters.

location	mean flow, 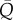 (ml s^−1^)	source/expression	reference
inlet	134.5	2D PC-MRI	n.a.
RCC	12.7	2D PC-MRI	n.a.
LCC	8.9	2D PC-MRI	n.a.
RSA/LSA	9.8	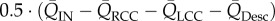 ^a^	n.a.
CT	20.7	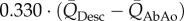 ^a^	[22]
SMA	14.0	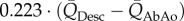 ^a^	[22]
RRA/LRA	14.0	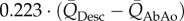 ^a^	[22]
AbAo	30.5	2D PC-MRI	n.a.

^a^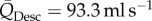, mean flow at the descending aorta obtained from 2D PC-MRI data (plane E in figure 1).

The workflow illustrated in figure 2*c* was followed, as described below.

(1) A WK3 analogue of the entire vascular system was used to determine the total system *C*^sys^, following the method used by Les *et al*. [23]. WK3 parameters 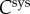, 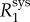 and 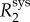 (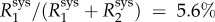 [23]) were iteratively varied to obtain the target *P*_sys_ and *P*_dia_ values, using as input *Q*_IN_(*t*). The obtained *C*^sys^ was equal to 0.99 ml mmHg^−1^.
(2) A 0D model representing the vascular system (figure 2*b*) was employed to tune *R*_1_, *R*_2_ and *C* of each WK3 coupled to the 3D aorta, considered *rigid* at this stage. The aorta was divided into segments modelled as 0D building blocks made by an *inertance* and a *resistance*, as shown in figure 2*b*.
  The compliance *C^i^* was calculated by distributing *C*^sys^ among each WK3 *i* proportionally to 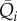. The ratio *R*_1_-to-*R*_tot_ (with *R*_tot_ = *R*_1_ + *R*_2_) was set following Les *et al*. [23], and 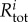 was calculated as 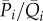, where 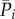 is the mean pressure at WK3 *i* obtained with the 0D model. 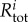 was adjusted to obtain the target flow distribution.
  The 0D model equations were solved with a backward-differentiation scheme using the software 20-sim (Controllab Products B.V., Enschede, The Netherlands).
(3) A CFD simulation (*rigid* 3D aorta plus 0D WK3 models) was run using the WK3 parameters from step 2.
  The systolic–diastolic pressure variation (Δ*P*, obtained from CFD simulations) and ΔA* (extracted from 2D cine-MRI sequences—equation (2.1)) were used to estimate the distensibility of various aortic segments (aortic arch, ascending, descending and abdominal aorta) as 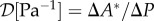. In the aortic segments presenting the dissection, both the TL and FL cross-sectional areas were considered in the calculation of the distensibility, as in [24].
  The distensibility of the main aortic branches was calculated from the pressure wave velocity (PWV) as 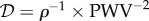 using the empirical relationship between PWV (m s^−1^) and the vessel diameter (*d* (mm)) from Reymond *et al*. [25],2.5
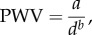
where *a* and *b* are 13.3 and 0.3.
  Finally, *C*_A_ (figure 2*a*) was calculated as 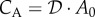, where *A*_0_ is the local vessel cross-sectional area. By integrating *C*_A_ along the vessel length, the compliance associated with the 3D aortic model was obtained (*C*^aorta^ = 0.41 ml mmHg^−1^).
(4) The parameters of the WK3 models coupled to the *compliant* aorta needed to be re-tuned to obtain physiological pressure and flow waveforms. Only the compliance attributed to the peripheral circulation (*C*^per^ = *C*^sys^ − *C*^aorta^) was distributed among the WK3 models. Although *R*_tot_ was kept equal to the one estimated at step 2 for each WK3, *R*_1_ was set equal to *ρ* × PWV/*A*_0_ (i.e. the characteristic impedance of the coupled 3D vessel) in order to reduce the impedance mismatch between the 3D compliant model and the 0D WK3, and thus to minimize the intensity of the non-physiological waves reflected by the Windkessel models [18,25]. WK3 parameters used for the multi-scale model (*compliant* 3D aorta plus 0D WK3 models) are reported in table 2.

**Table 2.** RCR parameters used for the Windkessel models coupled to the 3D compliant model as a result of the tuning procedure.

outlet	*R*_1_ (mmHg s ml^−1^)	*R*_2_ (mmHg s ml^−1^)	*C* (ml mmHg^−1^)
RSA	1.660	9.977	0.051
LSA	1.518	10.110	0.051
CT	1.588	3.858	0.107
SMA	1.488	6.604	0.072
RRA	2.947	5.102	0.072
LRA	2.162	5.919	0.072
AbAo	0.115	3.608	0.157

#### 2.3.4. Numerical simulations and post-processing

Simulations were run until reaching a periodic steady state. For the multi-scale model, this was achieved within three cardiac cycles after appropriate initialization; the last cycle was used for the analysis of results. Post-processing was done using CFD-Post (ANSYS Inc.) and Matlab (Mathworks, MA, USA). Time-averaged haemodynamic indices, such as time-averaged wall shear stress (TAWSS) and oscillatory shear index (OSI), were calculated according to Gallo *et al*. [26].

#### 2.3.5. Mesh

The extracted AD geometry (figure 2) was discretized with ICEM-CFD (ANSYS Inc.), adopting a tetrahedral mesh in the core region and seven prism layers at the walls (external aortic wall and IF sides). The resulting mesh consisted of about 506 000 elements, comparable to the grids used in other CFD studies of the aorta [2,14].

In order to select the computational grid, a preliminary mesh independence study was carried out using the rigid model on two additional grids, with 267 000 and 1 045 000 elements. Comparison between pressure and flow waves obtained at the outlets with the three meshes (coarse, medium and fine) showed a maximum difference of 4.8% and 2.5% for flow and pressure, respectively, between the coarse and medium mesh, and only 1.1% and 0.7% when comparing the medium and fine mesh. Thus, the medium mesh was deemed good enough for simulation purposes. The effect of the mesh on the results was further analysed for the compliant model on the coarse and medium grids. The results showed a maximum difference of 1.9% and 1.3% for the outlets' flow and pressure waves, respectively. The obtained TAWSS and OSI distributions over the aortic wall compared well qualitatively (electronic supplementary material). The difference between the mean and peak TAWSS and OSI was quantified over selected regions of interest (i.e. areas around the entry tear and FL wall, as shown in the electronic supplementary material), resulting in maximum differences of 7.6% and 5.8% for the peak and mean TAWSS, respectively, and 6.0% and 10% for the peak and mean OSI, respectively. These differences give an upper bound to the errors introduced by using a medium, rather than a fine, mesh in the model.

## 3. Results

### 3.1. Flow rate and pressure waveforms

**Figure 3.**
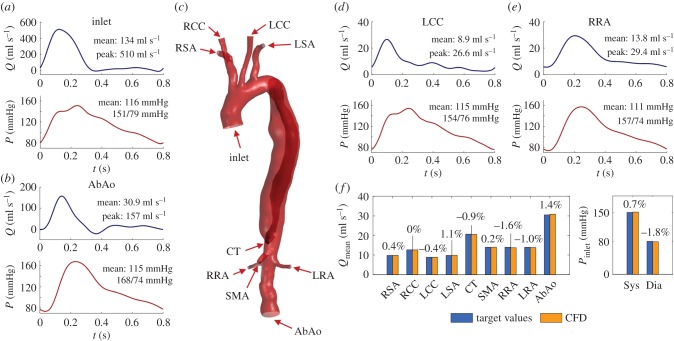
(*a*,*b*,*d*,*e*) Flow (*Q*) and pressure (*P*) waves obtained with CFD simulation at the inlet and selected outlets (*c*) of the compliant model. *P* is calculated as the average pressure over the boundary area. (*f*) Comparison between the flow and pressure target values used for the tuning procedure of the Windkessel parameters and CFD results. The relative percentage difference between CFD results and target values are reported in the figure, showing a good agreement. (Online version in colour.)

Comparisons between *target* pressure and mean flow values and simulation results are shown via histograms in figure 3 with good agreement between the two datasets (maximum difference of 1.8%).

Flow rates and pressure waveforms obtained with the multi-scale compliant model at the inlet and at selected outlets are shown in figure 3. Pressure waveforms from the aortic root (inlet) to the periphery (AbAo) exhibit typical physiological features, such as peripheral amplification of systolic and pulse pressures, and increase in the wave-foot time delay (i.e. point of lowest pressure). The PWV in the aorta was evaluated with the foot-to-foot method (9.21 m s^−1^), in consonance with PWV findings reported by Taviani *et al*. [27] for elderly people (i.e. 60–80 years).

In agreement with the literature [23], there is no backflow in the renal arteries, whereas a slight retrograde flow occurs at the abdominal aorta at early diastole.

These results suggest that the coupled 3D/0D, compliant and finely tuned model can correctly represent the effects of wave propagation along the vessel.

### 3.2. Aortic dissection haemodynamics

**Figure 4.**
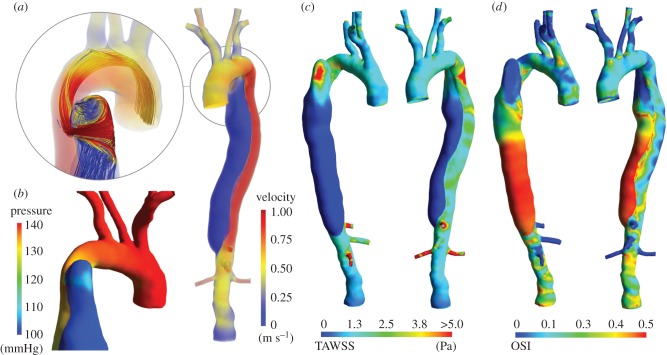
(*a*) Velocity magnitude at peak systole (*t* = 0.12 s). Inset: streamlines passing through the entry tear at peak systole showing the blood flowing into the FL. (*b*) Colour map of the pressure at peak systole in the ascending aorta and aortic arch. An area of increased pressure can be noted in the proximal FL where the blood flow impinges on the vessel wall. (*c*) Colour map of the TAWSS. (*d*) Colour map of the OSI.

Flow characteristics at peak systole are illustrated in figure 4*a*,*b*. Figure 4*a* shows the velocity magnitude in the 3D domain. High blood velocities (greater than 1.0 m s^−1^) can be seen in the TL, where the dissection causes a reduction in cross-sectional area, and in the coeliac trunk and renal arteries, due to low lumen area and high blood flow demand. Velocities in the FL are low in the medial/distal regions (less than 0.05 m s^−1^), due to the absence of secondary communications between the TL and FL. Nonetheless, a region of high velocity in the proximal part of the FL is observed; during systole, a net flow is drawn through the small entry tear into the FL due to the pressure gradient between the TL and the FL (figure 4*b*) and the dilation of the vessel caused by the increasing pressure. At peak systole, the flow is well organized throughout apart from the proximal FL, where a high-velocity jet-like flow, rolling up to form a vortex, is observed (figure 4*a*).

Blood flowing through the entry tear impinges on the FL wall causing a localized pressure increase (figure 4*b*), potentially leading to further enlargement/rupture of the already structurally compromised FL wall.

TAWSS and OSI distributions are shown in figure 4*c*,*d*. High TAWSS values (greater than 5 Pa) are observed in the region around the entry tear, which is coherent with the flow description presented previously. Very low TAWSS is seen in the medial/distal part of the FL due to the almost stagnant flow obtained here.

Regions with moderate OSI are observed throughout the aorta (figure 4*d*); although the flow is well organized in systole, it is highly disorganized in diastole. OSI values in the aortic branches are low because the flow is anterograde throughout the cardiac cycle here. Instead, a very high OSI is reported in the distal part of the FL due to the flow wave's biphasic nature in this region, caused by the alternating expansion and contraction of the FL during systole and diastole (as described in §3.6 for the compliant model).

### 3.3. Comparison between two-dimensional phase-contrast MRI data and computational fluid dynamics results: flow rate in the dissected aorta

**Figure 5.**
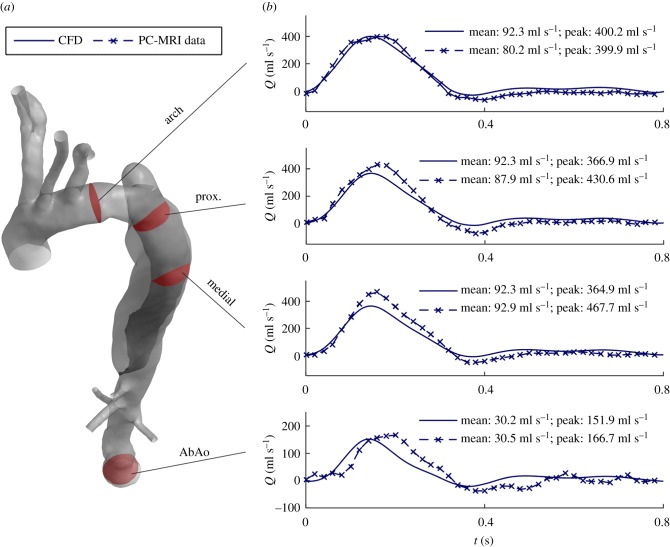
Comparison between computed and measured flow rates at different locations along the dissected aorta, as indicated in (*a*). (*b*) CFD results are presented with the solid lines, while 2D PC-MRI data are shown by the dashed lines. (Online version in colour.)

Figure 5 compares the blood flow rate measured with PC-MRI and obtained with the CFD model at four different locations: the arch (distally to the LCC), two sections of the TL in the descending aorta, and the abdominal aorta. Peak flow rates are underestimated by the CFD model, with a relative difference between measured and computed flows of 0.1%, −15%, −22% and −9%, respectively. Overall, predicted waveforms agree with those measured *in vivo*, with a good synchronization between the two waves in the first three locations, even if a delay is present in the AbAo.

### 3.4. Vessel wall displacement: comparison between computational fluid dynamics results and two-dimensional cine-MRI data

**Figure 6.**
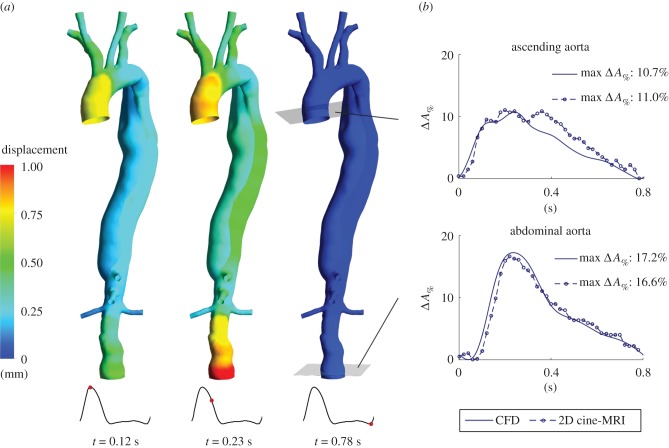
(*a*) Displacement of the vessel wall obtained with the computational model at peak systole (*t* = 0.12 s), systolic deceleration phase (*t* = 0.23 s) and end-diastole (*t* = 0.78 s). (*b*) Percentage cross-sectional area variation (Δ*A*_%_) during a cardiac cycle at the level of the ascending and abdominal aorta: comparison between CFD results and 2D cine-MRI measurements. Δ*A*_%_ = (*A* – *A*_0_)/*A*_0_, where *A* is the cross-sectional area of the vessel lumen and *A*_0_ is the lowest cross-sectional area of the vessel lumen throughout a cardiac cycle.

Blood pressure changes during the cardiac cycle drive aortic dilation and contraction. At peak systole, the maximum displacement relative to the undeformed (i.e. diastolic) configuration predicted by the simulations is localized in the ascending aorta (approx. 0.74 mm). Subsequently, the pressure peak reaches the AbAo at *t* = 0.23 s, with a maximum vessel expansion = 1.23 mm (figure 6*a*). The computed maximum percentage cross-sectional area variation (Δ*A*_%_) is equal to 10.7% and 17.2% at the ascending and abdominal aorta, respectively, and is in agreement with the values extracted from 2D-MRI data (11.0% and 16.6%, respectively; figure 6*b*). Moreover, the predicted Δ*A*_%_ waveforms compare well with the measured ones, suggesting that the MB method can reliably reproduce vessel expansion due to pressure changes during the cardiac cycle. The measured Δ*A*_%_ waveform at the ascending aorta presents a peak at early diastole, absent in the computed one, that may be due to the dicrotic wave, which is not completely resolved by the simulation.

The dicrotic notch in the pressure wave is caused by the abrupt change of blood flow due to the closure of the aortic valve. However, the flow-rate curve applied at the inlet of the CFD model, which was extracted from PC-MRI data, potentially affected by measurement inaccuracies, lacks such a rapid change at the beginning of the diastole, and thus the production of a dicrotic wave in the simulation may be affected.

### 3.5. True lumen–false lumen transmural pressure and intimal flap displacement

**Figure 7.**
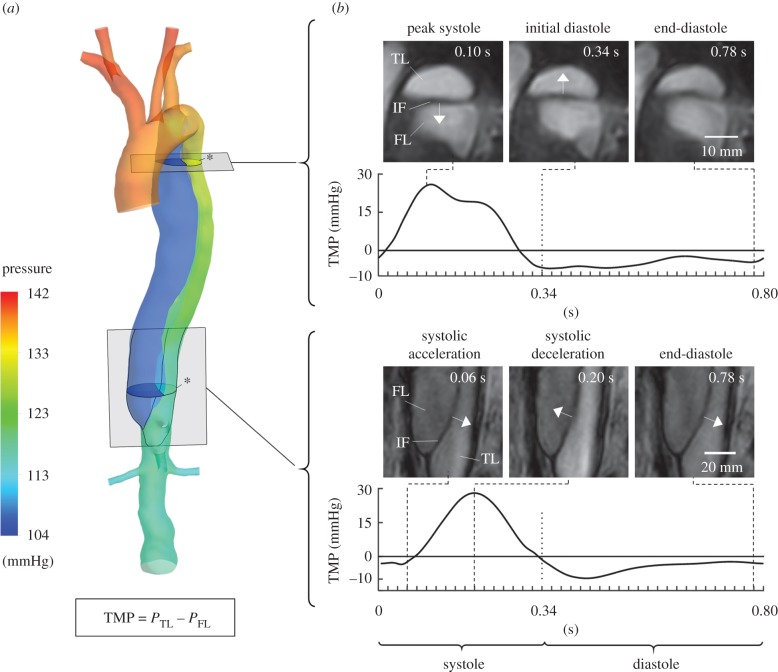
(*a*) Pressure colour map at peak systole (*t* = 0.12 s). (*b*) Comparison between the TMP across the IF obtained with the CFD compliant model during a cardiac cycle and the displacement of the IF observed from 2D cine-MRI data at different time instances (TMP = *P*_TL_ − *P*_FL_, where *P*_TL_ and *P*_FL_ are the pressures in the TL and FL averaged over a cross-section—indicated by an asterisk in the figure—located at the same level of the respective 2D cine-MRI slice). It can be noted that the direction of the IF displacement agrees well with the sign of the TMP: a positive TMP corresponds with the motion of the IF through the FL, whereas a negative TMP relates to the motion of the IF through the TL.

Pressure differences between the TL and the FL can provide insight into the actual state and probable progression of the dissection but cannot usually be assessed in the clinic. For instance, high pressure in the FL can lead to further expansion, or even rupture of the FL, while causing a narrowing of the TL [4]. Simulated pressure distributions at peak systole (figure 7*a*) show higher pressure in the TL than in the FL. The temporal variation of the transmural pressure across the IF (TMP = *P*_TL_ − *P*_FL_, where *P*_TL_ and *P*_FL_ represent the pressure in the TL and FL, respectively) at proximal and distal levels is shown in figure 7*b*. The TMP curves are compared with 2D-MRI sequences at the same locations showing the IF displacement during the cardiac cycle. The TMP is positive (i.e. pressure higher in the TL) during most of systole at both locations, reaching a value of about 30 mmHg, with the distal TMP curve delayed with respect to the proximal one. Instead, a negative TMP (i.e. pressure higher in the FL) is found throughout diastole with a minimum value around −10 mmHg. Comparison of TMP curves with MRI sequences shows that the IF displacement direction agrees well with the computed TMP: a positive TMP corresponds with the motion of the IF through the FL; conversely, a negative TMP relates to the motion of the IF through the TL, indicating reliability of the predicted pressure field while providing further validation of the methodology presented herein.

### 3.6. Comparison between rigid and compliant model

**Figure 8.**
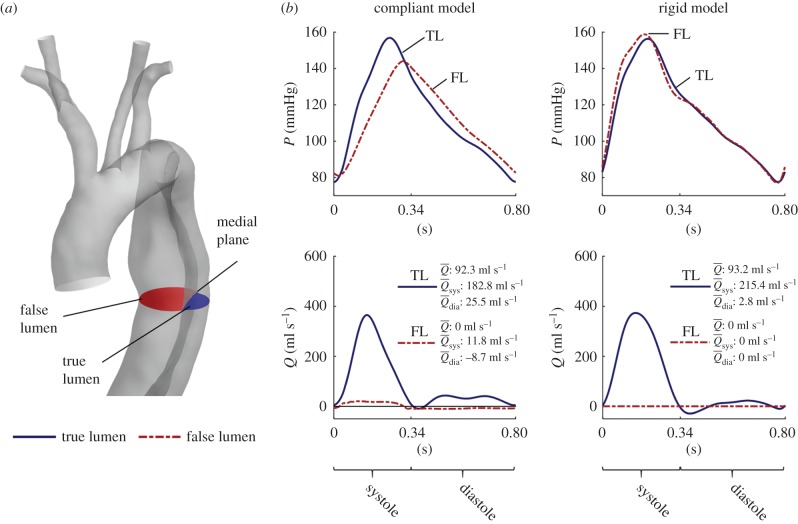
(*a*,*b*) Comparison between CFD results obtained with compliant and rigid models. TL results are presented with *solid blue lines*, whereas FL results are shown by *dashed red lines*. The pressure results are presented as the averaged values calculated over the cross-sections shown in the schematic in (*a*), whereas the flow results are calculated as the integral of the velocity over the same sections (a positive flow corresponds to fluid moving towards the abdominal aorta). 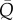, 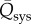 and 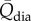 are the mean flow rates calculated over the entire cardiac cycle, the systole and diastole phase, respectively. (Online version in colour.)

Figure 8 compares the flow rate and pressure waves of a rigid versus a compliant model at a medial section of the dissection. It should be noted that, even if pressure values obtained by both models are of the same order of magnitude, the relative pressure in the two lumina follows an opposite trend: the compliant model predicts higher pressures during systole and lower pressures during diastole in the TL than in the FL, while the opposite is found with the rigid model. As previously discussed, the available clinical dataset indicates that the correct pressure distribution is the one computed by the compliant model, not the rigid one.

Additionally, while the cycle mean flow rate (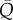) is approximately the same in both models, its distribution among the cardiac phases is different: the compliant model predicts a lower TL flow during systole and a higher TL flow during diastole than the rigid model. Owing to the aortic compliance, the vessel can store blood during systole and release it during diastole. Following this, 28 ml of blood is accumulated in the aorta during systole due to a pressure increase of 68 mmHg. Consequently, peak flow rates in the compliant model are less pronounced than those obtained with the rigid model. As expected, the flow rate in the FL computed by the rigid model is equal to zero throughout the cardiac cycle. On the other hand, the compliant model predicts a small but non-negligible net flow in the FL resulting from the alternate dilation and contraction of the FL during systole and diastole, respectively.

**Figure 9.**
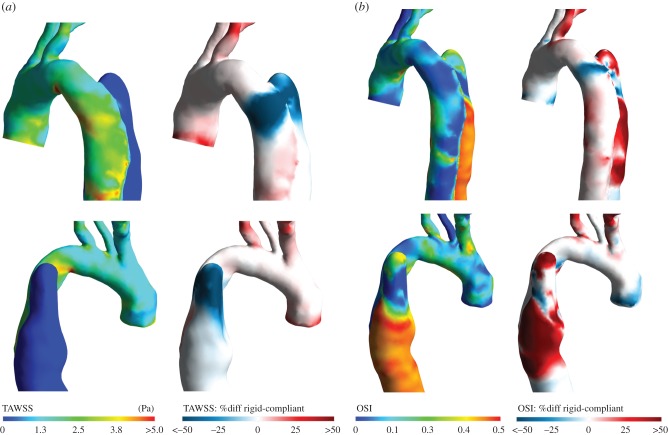
Haemodynamic indices in the ascending aorta and aortic arch obtained with the rigid CFD model, and comparison with compliant model results. (*a*) TAWSS obtained with the rigid model and percentage difference relative to the compliant model results, calculated as: %diff = (TAWSS_R_ − TAWSS_C_)/(5 (Pa)). (*b*) OSI obtained with the rigid model and percentage difference relative to the compliant model results, calculated as: %diff = (OSI_R_ − OSI_C_)/0.5 (where OSI_R_ and OSI_C_ are the OSI values for rigid and compliant, respectively). The percentage difference figures were normalized by values representing the range of the respective parameter distributions for better visualization.

The dissimilar haemodynamics computed by the two models results in different TAWSS distributions; this is particularly true in the region around the entry tear, an important indicator of disease progression. Figure 9*a* shows the TAWSS distribution obtained with the rigid model (TAWSS_R_) and the relative difference with the one computed by the compliant model (TAWSS_C_). In the proximal part of the FL, TAWSS_R_ is significantly lower than TAWSS_C_. The OSI distributions obtained with the two models also differ considerably (figure 9*b*).

## 4. Discussion

The present work presents a significant advance of the current state of the art in CFD simulations of AD for clinical support. The vast majority of computational studies in the literature are limited by simplified assumptions about the treatment of BCs and wall motion.

For instance, despite the fact that the cardiac output is patient specific and known to be affected by medical treatment [10], due to the lack of *in vivo* data, flow waves of healthy subjects examined in published studies are commonly employed as inflow BCs [12]. Overly simplified assumptions are often made regarding outflow BCs. A flow split is commonly adopted to prescribe flow to the supra-aortic branches, by diverting 5% of the total inlet flow into each branch during the entire cardiac cycle [12]. This proportion, reported for the healthy human aortic system [12], may be different in pathological cases. Moreover, this imposes a constant flow diversion into each branch that can have a major impact on wall shear stress (WSS)-based indices, as shown by Gallo *et al*. [26]. Additionally, zero-pressure conditions are often used as outflow BCs at the abdominal aortic branches [11]. Although this approach is suitable for rigid models with a single outlet, it is inadequate for multi-branched or deformable models as flow distribution among the vascular branches strongly depends on the downstream vasculature. To overcome these limitations, 0D WK3s have been used as BCs in more advanced AD simulations [2,5].

Finally, a rigid-wall approximation is commonly adopted. However, as shown in our previous work [14] and also confirmed here, vessel wall motion has a significant impact on clinically relevant haemodynamic markers for AD. FSI methods, coupling CFD with finite-element modelling of the aortic wall, are subject to uncertainties regarding the mechanical properties of the tissue, which remain unknown for the dissected aorta and may be patient specific [13]. The high computational cost of FSI simulations can also be prohibitive in the context of CFD models for medical support. Thus, simpler methods to account for wall motion in such applications are necessary.

In this study, we developed a patient-specific, compliant CFD model of a chronic type B AD with patient-specific tuned dynamic BCs. A rich *in vivo* dataset comprising multiple non-invasive imaging modalities was used to inform and validate the computational model. A fast procedure for BC tuning and a new, efficient method to model the wall motion have been proposed to tackle some of the aforementioned challenges.

The model was tuned using brachial blood pressure measurements and flow and displacement data obtained via MRI. The parameters of the WK3 models coupled to 3D models of the dissected aorta were successfully set, and target values specified on mean flows at model outlets and on blood pressure at the inlet were met. The aortic wall distensibility/compliance was estimated indirectly from cross-sectional vessel area and pressure changes at different sections along the aorta. At the level of the dissected descending aorta, a distensibility of 0.42 × 10^−5^ Pa^−1^ was found; this is comparable to values reported by Ganten *et al*. [24] for a cohort of patients with chronic type B AD. This value is significantly lower than that for healthy patients of the same age [24].

The proposed MB method allowed us to account for aortic wall deformation and compliance in the CFD simulation. A linear, elastic relationship was assumed between wall displacement and fluid forces. Although it is well known that the stress–strain relationship of the vessel wall is generally nonlinear, experimental evidence suggests that the assumption of a linear constitutive relation for the arterial wall is justified in the range of physiological pressures [28]. It has been shown that the effects of nonlinearity and viscoelasticity on pressure and flow waves are minor in the aorta [25,29]. Also, limited data availability on the viscoelastic properties of the arterial wall in the literature [25] implies that using more advanced constitutive relations would introduce additional complexity and parameter uncertainties.

The MB method allowed us to capture the essential physics of arterial deformation and wave propagation, as shown in the Results section.

The compliant model simulation took 13 h for one cardiac cycle on a desktop computer (Intel Xeon E5, 8 cores, 32 GB RAM) and this is a significant improvement over FSI simulations. As an example, the computational time reported in our previous work for an FSI simulation of AD was 57 h per cycle [14]. In this context, the MB algorithm is much less computationally expensive and easier to implement, and this is critical for clinical use. Moreover, the model can be easily tuned with patient-specific vessel motion data obtained non-invasively in the clinic (via 2D cine-MRI). This represents an advantage over previous AD studies using traditional FSI methods [14], where uncertainties related to material properties, which are often taken from the literature, can be considerable.

Comparison between computed and measured blood flows at different sections in the descending aorta showed good agreement; modelling the compliance of the aortic wall allowed amplitude and phase discrepancies between measured and simulated waveforms observed in previous rigid AD studies [5] to be reduced. Therefore, it appears clear that introducing the compliance of the vessel wall in the CFD model allows a better replication of the *in vivo* fluid dynamics situation, and represents an improvement of this study compared with previous rigid AD models [2,5].

It should be noted that there is still a difference between the CFD results and PC-MRI, which may be attributed to factors related to the *in vivo* data, such as inaccuracies in the segmentation of the lumen contours on magnetic resonance images, noise and the spatial resolution of PC-MRI, and to the model limitations discussed below. The under-estimation of peak flow rates in the TL can affect the derived fluid-dynamic variables, such as TAWSS and OSI, and potentially lead to an under-estimation of the WSS in this region. However, the discrepancies reported here are smaller than those obtained in other fully rigid AD models, where differences of up to 56% [5] and 28% [11] at peak flow rate are present.

The results also show that the use of a rigid-wall approximation requires careful consideration. For instance, the TMP between the two lumina predicted by a *rigid model does not agree* with the magnetic resonance images. TMP is an important physiological variable that cannot be measured *in vivo* non-invasively and without risk for the patient, but can be predicted instead by CFD simulations; *hence*, *accurate prediction is essential in order to use this parameter in the clinic*. The compliant model predicted higher systolic pressure in the TL, and higher diastolic pressure in the FL; these findings compare well with experimental results obtained by Tsai *et al*. [30] with a model of a chronic type B AD lacking a distal re-entry-tear, similar to the one studied here. Higher diastolic FL pressure may lead to an increased aortic wall tension and consequent risk of lumen expansion.

TAWSS is thought to be important for AD initiation, affecting growth and enlargement of the FL [2]. The high TAWSS found by the compliant model in the entry-tear region can be a predictor of further enlargement. It has been recently reported by Doyle & Norman [4] that areas of low TAWSS may be correlated to regions of rapid local expansion in type B AD. In the present study, the medial and distal parts of the FL exhibit low TAWSS and high OSI, which may indicate elevated risk.

### 4.1. Limitations

Owing to the difficulties in acquiring *in vivo* haemodynamic data using non-invasive methods, some model parameters were specified using non-patient-specific information; these include the *R*_1_-to-*R*_tot_ ratio used to calculate the WK3 parameters coupled to the aortic rigid model, and the population-based law (equation (2.5)) used to estimate the PWV in the aortic branches. Moreover, the flow distribution among the abdominal branches was specified based on data taken from the literature for healthy patients. Although this assumption holds for the subject studied in this paper, in which the visceral branches were not involved in the dissection, it may not be valid in general. For AD cases in which the flow into these arteries is expected to be impaired, it may be useful to acquire additional flow data at the coeliac and infrarenal regions to inform the model. However, for the patient studied, these assumptions allowed us to obtain physiological pressure and flow waves, consistent with the available clinical data.

This study assumes that the IF behaves like a rigid zero-thickness membrane separating the two lumina as the resolution of CT images did not allow the IF thickness to be estimated with reasonable accuracy. The IF thickness estimated from MRI slices was about 2 mm, which is small compared with the diameter of the aorta; hence, the use of this approximation was deemed appropriate. Moreover, the IF of chronic type B AD is expected to be stiff and not as compliant as in acute settings [30]; this was confirmed by the cine-MRI sequences showing relatively small displacement of the IF (approx. 3 mm maximum displacement in the proximal and distal parts, and less than 1 mm in the central segment of the flap). However, in the regions where the IF displacement is at its maximum, the fluid flow can be significantly affected by the flap motion, and the assumption of a rigid and zero-thickness IF may be the cause of the discrepancy observed between the computed and measured peak flow rates at the locations where the TL and FL coexist. Further work will examine the effect of IF motion on blood flow distribution and pressure in the TL and FL.

## 5. Conclusion

This paper presents a novel, patient-specific multi-scale modelling approach coupled to Windkessel BCs to study AD. The approach accounts for wall compliance in a computationally efficient manner not explored hitherto. Simulation results were compared with patient-specific clinical data as part of a rich dataset from multiple non-invasive imaging modalities, comprising haemodynamics and vessel motion data, and covering the entire extension of the dissection. This work, at the interface of medicine and engineering, was developed in close collaboration with clinicians, who have stressed that the relative good agreement achieved between simulations and the *in vivo* data demonstrates that the proposed approach can successfully capture the haemodynamics in a chronic type B AD and can potentially lead to a powerful decision-making tool for the clinical management of AD. For instance, the model can be used to simulate different interventional strategies (e.g. covering of the entry tear with a stent graft, fenestration of the IF) or medical treatments (e.g. β-blocker therapy) and analyse their effects on the fluid dynamics in the dissection (in particular pressure and blood flow in the FL). Further research from the authors will include the application of the tool on a small cohort of patients. This is work that is well under way with data already collected for a number of patients.

## Supplementary Material

Supplementary Material List

## Supplementary Material

Fig 1-PCMRI

## Supplementary Material

Fig 3

## Supplementary Material

Fig 5

## Supplementary Material

Fig 6

## Supplementary Material

Fig 7

## Supplementary Material

Fig 8

## Supplementary Material

Mesh Sensitivity

## Data accessibility

The patient-specific geometry of the dissected aorta used in the CFD model is available in .stl format on the Digital Repository Figshare: doi:10.6084/m9.figshare.5354359.v1 [15]. Additional supporting data are available as part of the electronic supplementary material.
