# Supplementary material for: Computational tools for clinical support: a multi-scale compliant model for haemodynamic simulations in an aortic dissection based on multi-modal imaging data
Source: J R Soc Interface. 2017 Nov 8;14(136):20170632. doi: 10.1098/rsif.2017.0632 (PMC5721167; doi:10.1098/rsif.2017.0632)
Supplement: Supplementary Material List [file rsif20170632supp1.pdf]

Electronic Supplementary Material List

- **Numerical data of the in vivo flowrate waveforms in Figure 1.**: Blood flow waveforms measured with PC-MRI at the locations indicated in Fig. 1b. In the compressed folder, there are seven Comma Separated Values files, one for each curve in Fig. 1c. Each file contains two columns: the first column contains the time [s], the second contains the blood flowrate [ml/s].

- **Numerical data of the graphs in Figure 3**: The compressed folder contains five Comma Separated Values files with the numerical data of Fig. 3.

- **Numerical data of the CFD flowrate waveforms in Figure 5**: The compressed folder contains four Comma Separated Values files with the numerical data of the CFD flowrate waveforms reported in Fig. 5.

- **Numerical data of the area variation waveforms in Figure 6**: The compressed folder contains four Comma Separated Values files with the numerical data of the cross-section area variation waveforms reported in Fig. 6.

- **Numerical data of the transmural pressure waveforms in Figure 7**: The compressed folder contains two Comma Separated Values files with the numerical data of the transmural pressure waveforms reported in Fig. 7.

- **Numerical data of the graphs in Figure 8**: The compressed folder contains eight Comma Separated Values files with the numerical data of Fig. 8.

- **Mesh Sensitivity Study Results:** Comparison between the time-averaged wall shear stress (TAWSS) and oscillatory shear index (OSI) distributions obtained with the multiscale compliant model using the coarse and medium grids.
