# Supplementary material for: Computational tools for clinical support: a multi-scale compliant model for haemodynamic simulations in an aortic dissection based on multi-modal imaging data
Source: J R Soc Interface. 2017 Nov 8;14(136):20170632. doi: 10.1098/rsif.2017.0632 (PMC5721167; doi:10.1098/rsif.2017.0632)
Supplement: Mesh Sensitivity [file rsif20170632supp8.pdf]

Supplementary Material - Mesh Sensitivity Study

Within the mesh sensitivity study, the mesh effects on the time-averaged wall shear stress (TAWSS) and oscillatory shear index (OSI) distributions obtained with the multiscale compliant model were evaluated.

In Fig. S1 and S2 are shown the TAWSS and OSI colormaps computed on the aortic wall using two computational grids: a coarse and a medium mesh.

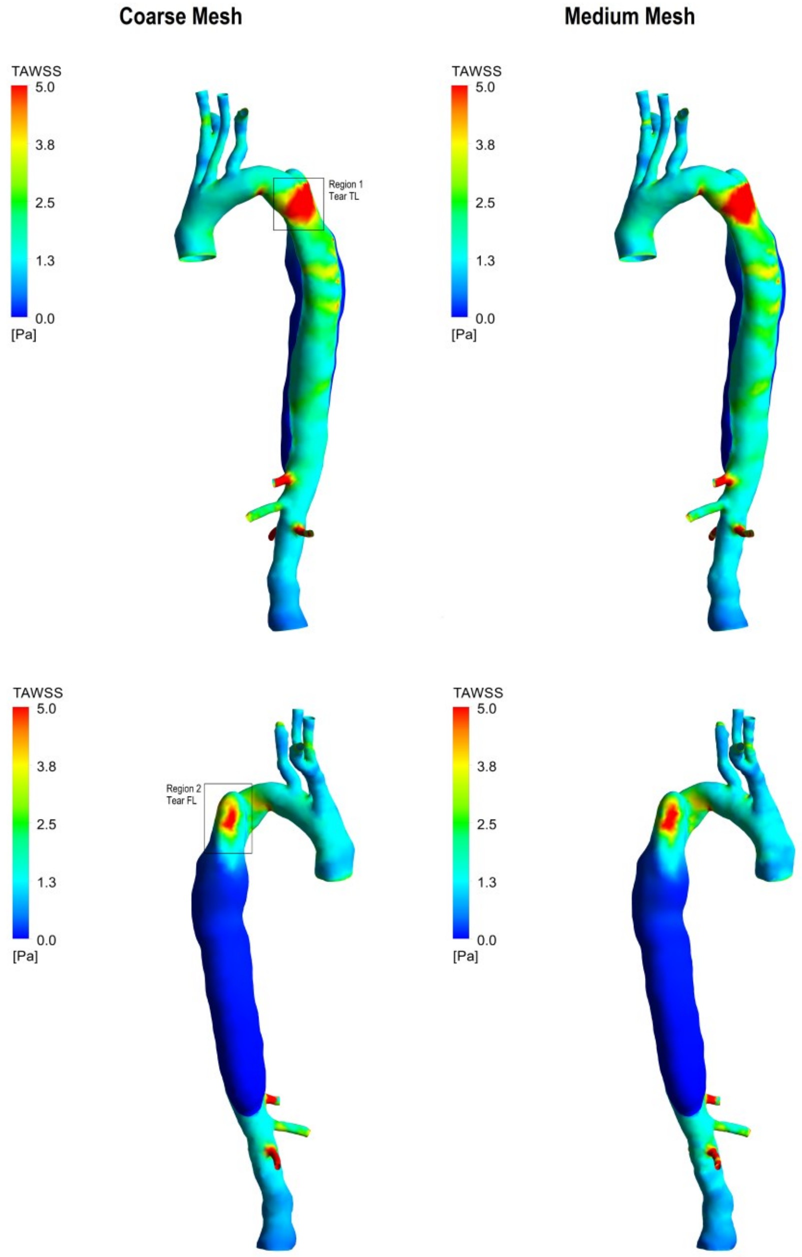

Fig. S1 Time-averaged wall shear stress distributions obtained with the compliant model using the coarse (left) and medium mesh (right).

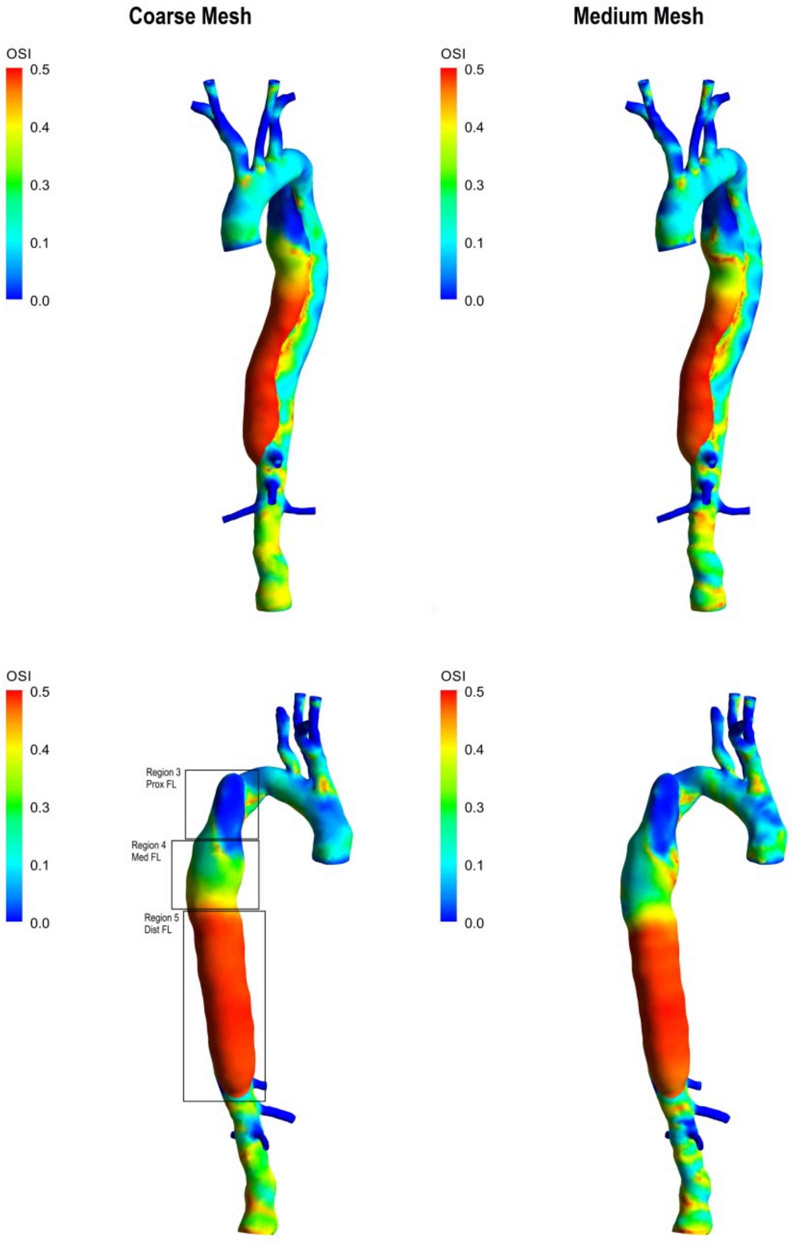

Fig. S2 Oscillatory shear index distributions obtained with the compliant model using the coarse (left) and medium mesh (right).

The differences between the mean and peak TAWSS and OSI over selected regions of interest (indicated in Fig. S1 and S2) were quantified and are reported in Table S1.

Table S1: Comparison between TAWSS and OSI obtained with the coarse and medium mesh.

Region	Index	Coarse	Medium	Err. % *
Region 1 - Tear TL	TAWSS_mean [Pa]	4.96	5.14	-3.45
	TAWSS_peak [Pa]	22.98	21.36	7.56
Region 2 - Tear FL	TAWSS_mean [Pa]	2.68	2.85	-5.76
	TAWSS_peak [Pa]	5.97	6.31	-5.47
Region 3 - Prox. FL	OSI_mean [ ]	0.057	0.058	-0.20
	OSI_peak [ ]	0.458	0.488	-6.00
Region 4 - Med. FL	OSI_mean [ ]	0.266	0.215	10.20
	OSI_peak [ ]	0.492	0.490	0.40
Region 5 - Dist. FL	OSI_mean [ ]	0.482	0.473	1.80
	OSI_peak [ ]	0.498	0.498	0.00

* $Err.\%_{TAWSS} = (TAWSS_{coarse} - TAWSS_{medium})/TAWSS_{medium}$
$Err.\%_{OSI} = (OSI_{coarse} - OSI_{medium})/0.5$
